# Supplementary material for: Experience Measures after Radical Prostatectomy: A Register-Based Study Evaluating the Association between Patient-Reported Symptoms and Quality of Information
Source: Healthcare (Basel). 2022 Mar 12;10(3):519. doi: 10.3390/healthcare10030519 (PMC8953280; doi:10.3390/healthcare10030519)
Supplement: Supplementary file 1 [file healthcare-10-00519-s001.zip › healthcare-1605297-supplementary.pdf]

healthcare

MDPI

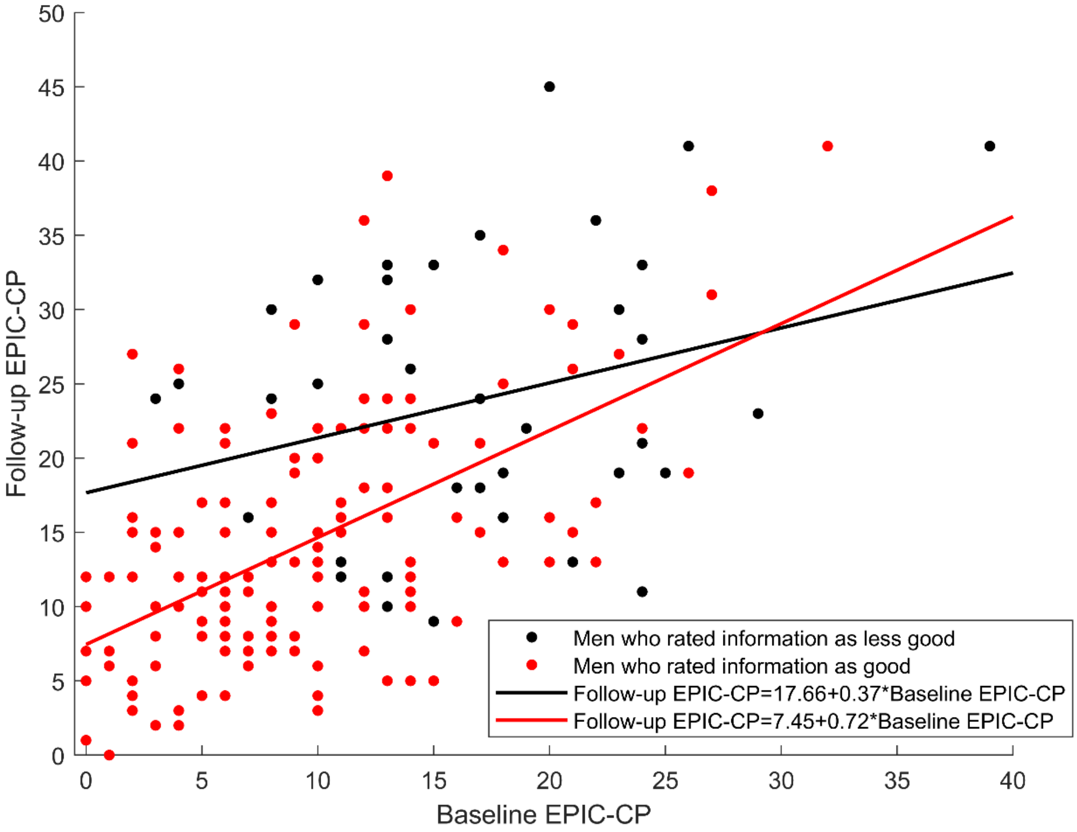

Figure S1. Scatterplot showing the association between EPIC-CP at baseline and follow-up, stratified on how information was rated on the item "I received good information about adverse effects".
